# Numerical Modeling of Toe-to-Heel Air Injection and Its Catalytic Variant (CAPRI) under Varying Steam Conditions

**DOI:** 10.1021/acs.energyfuels.2c03069

**Published:** 2022-12-22

**Authors:** Thomas Lopeman, Hossein Anbari, Gary Leeke, Joseph Wood

**Affiliations:** † School of Chemical Engineering, University of Birmingham, Edgbaston, BirminghamB15 2TT, U.K.; ‡ Faculty of Engineering, University of Nottingham, NottinghamNG7 2RD, U.K.

## Abstract

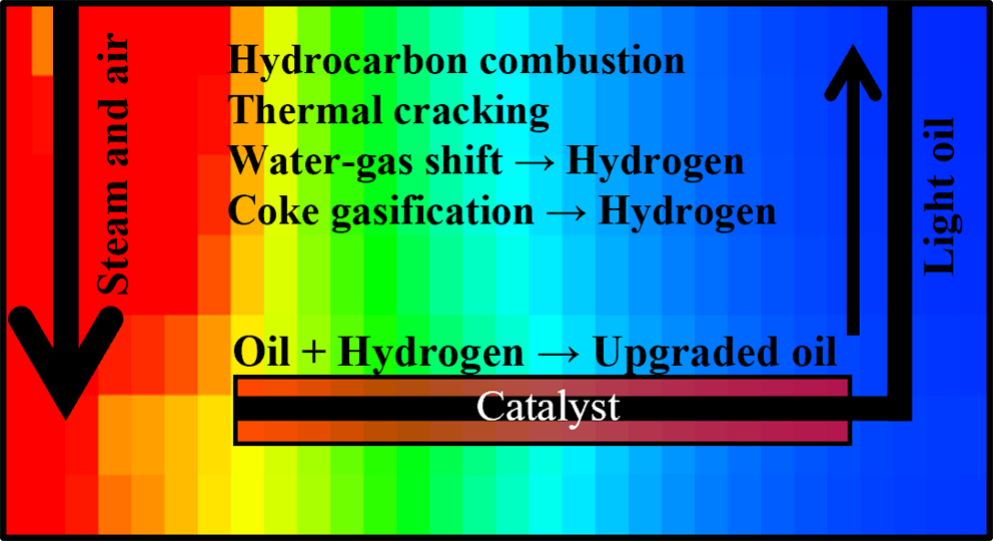

There are huge reserves of heavy oil (HO) throughout the world that can be energy-intensive to recover. Improving the energy efficiency of the recovery process and developing novel methods of cleaner recovery will be essential for the transition from traditional fossil fuel usage to net-zero. In situ combustion (ISC) is a less used technique, with toe-to-heel air injection (THAI) and catalytic processing in situ (CAPRI) being specialized novel versions of traditional ISC. They utilize a horizontal producing well and in the case of CAPRI, a catalyst. This paper aims to investigate the impact that injected steam has on both the THAI and CAPRI processes for the purpose of in situ HO upgrading and will help to bridge the gap between the extant laboratory research and the unknown commercial potential. This study also presents a novel method for modeling the THAI–CAPRI method using CMG STARS, proposing an in situ hydrogen production reaction scheme. THAI and CAPRI experimental-scale models were run under three conditions: dry, pre-steam, and constant steam. Starting from a reservoir American Petroleum Institute (API) of 10.5°, THAI reached an average API of ∼16 points, showing no increase in the API output with the use of steam injection. A decreased API output by ∼0.7 points during constant steam injection was achieved due to a high-temperature oxidation-dominant environment. This decreases the reactant availability for thermal cracking. The CAPRI dry run reached an API of 20.40 points and achieved an increased API output for both pre-steaming (∼21.17 points) and constant steaming (∼22.13 points). The mechanics for this increased upgrading were discussed, and catalytic upgrading, as opposed to thermal cracking, was shown to be the reason for the increased upgrading. Both processes produce similar cumulative oil (∼3150 cm^3^) during dry and pre-steamed runs, only increasing to ∼3300 cm^3^ with the constant steam injection during THAI and 3500 cm^3^ for CAPRI.

## 1 Introduction

Heavy oil (HO) and bitumen reserves are typically less preferable to lighter oil reservoirs, from which fuels can be extracted more efficiently at a lower cost. However, as these light oil (LO) reserves become more scarce or difficult to produce, the economical production of HO and bitumen reserves via environmentally acceptable techniques has attracted more attention. Hein^1^ estimates that 5.6 Gbbl of HO and bitumen can be found across the world, with the majority being found in Canada and Venezuela. HOs and bitumen are defined as having an American Petroleum Institute (API) gravity between 10 and 20° and less than 10°, respectively. With an ever-increasing demand to move toward carbon-neutrality and green energy sources, many methods of oil and gas production are quickly becoming obsolete.^2^ However, traditional fossil fuels are still necessary during the transition to renewable energy sources while working toward the 2050 net-zero Paris agreement.^3^ During this transition, it will become increasingly important to reduce the energy input required for the production and processing of the crude oils required.

Currently, the main methods of HO and bitumen recovery utilize steam with steam flooding and steam-assisted gravity drainage, considered the core techniques.^4^ Nevertheless, often, these methods rely on a very specific reservoir architecture (e.g., large pay zone thickness), making them very limited in their use.^5^ Additionally, these technologies have substantial imported energy requirements and emit considerable quantities of carbon dioxide (CO_2_) during steam production, alongside substantial heat loss from the wellbore, necessitating additional waste-water management. Most importantly, they have been shown to not deliver evident oil upgrading within the reservoir, meaning the production of low API hydrocarbons requiring additional operating costs per barrel for ex situ upgrading,^6^ alongside a further surface-level energy input.

In situ combustion (ISC) is used within Canada in HO and bitumen fields, whereby contemporaneous injection of oxygen-rich air and the heating of the injection well stimulates the combustion of the oil-originally-in-place. The combustion of the in situ hydrocarbons produces temperatures that induce cracking reactions, shortening the hydrocarbon chains.^7^ This cracking occurs just ahead of the combustion front, which can reach temperatures exceeding 400 °C.^8,9^ These shorter-chain hydrocarbons require less energy to produce and need less ex situ processing at surface facilities, which make them an economically preferable product. ISC also benefits from the avoidance of necessary steam injection during the recovery process as steam is generated within the reservoir as a result of the combustion temperatures heating the native water and combustion-produced water vapor.

Toe-to-heel air injection (THAI) is an ISC technique that utilizes a horizontal production well. A horizontal producer creates a short-distance displacement environment, eliminating the lag time between combustion initiation and oil production seen within traditional ISC. The thermally upgraded oil generated just ahead of the combustion front flows down the horizontal producer well via gravity and is produced rapidly. THAI also greatly reduces gas override where the injected gas used for combustion is produced over the oil after finding channels through the reservoir, avoiding reactions (i.e., oxygen production reducing in situ oxidation) and being produced.^10^ Gas override is a key indicator for the mechanical stability and efficiency of the ISC process, with higher production of injected gases having negative impacts on the production rates and total recovery.^5,11^ THAI has been implemented in semi-commercial projects within Canada^12,13^ with limited success thus far. The Kerrobert project underwent 10 years of combustion, utilizing the THAI process successfully, still producing upward of 100 bbl/day after 10 years of air injection.^12^ Despite the successful global field trials in Canada (Whitesands, Kerrobert, etc.), India (Balol and Lanwa), and China (Shuguang and Fengcheng),^12^ THAI is still largely not understood. The process has been shown in lab experiments to deliver considerable in situ oil upgrading, whereby the API has been shown to increase by 3–8°, dependent on the operating conditions.^14−18^

Catalytic processing in situ (CAPRI) is a catalytic add-on that can be combined with the THAI process (THAI–CAPRI) whereby an industrial hydroprocessing catalyst is packed along the horizontal producer well. The hydroprocessing catalyst [e.g., alumina-supported cobalt-oxide–molybdenum-oxide (CoMo/γ-Al_2_O_3_) or alumina-supported zinc-oxide–copper-oxide (ZnCu/γ-Al_2_O_3_)]^19^ facilitates the catalytic upgrading of heated oil through the hydrogenation, hydrotreating, and hydrocracking of the heated in situ HO with in situ-generated hydrogen. THAI–CAPRI was first introduced into the literature in the early 2000s^18,20,21^ and has undergone extensive laboratory testing since. Laboratory testing of THAI–CAPRI on HOs shows that the addition of a catalyst can increase the API upgrading by as much as 5° above that of just THAI.^14−18,21,22^

THAI–CAPRI did not undergo numerical modeling until Hasan and Rigby^23^ used CMG STARS, a thermal processes reservoir simulation software, to model laboratory-scale THAI–CAPRI utilizing the co-injection of air/oxygen and hydrogen. The injected hydrogen was used to represent the potential hydrogen produced through reactions such as water gas shift and coke gasification. Their study investigated the oil recovery potential of THAI–CAPRI under different catalyst packing porosities and hydrogen to air ratios, concluding that increased air to hydrogen ratios and increased catalyst packing porosity both positively influence the oil recovery of the THAI–CAPRI process. A follow-up study^19^ again used CMG STARS to numerically model THAI–CAPRI, this time investigating the impact of the operating pressure on the process’s oil upgrading ability. It was concluded that a higher operating pressure (8000 kPa) produces higher API oils (up to 25 API) and in larger quantities than lower operating pressures (500 kPa). To fully utilize THAI–CAPRI as a method of HO upgrading and production, it is first necessary to realize its predictive potential using accurate and reliable numerical modeling. Modeling to date has utilized injected hydrogen as a proxy for the hydrogen that would otherwise be created in situ during the THAI–CAPRI process. However, due to the real-life risks associated with co-injecting oxygen and hydrogen, all hydrogen within the THAI–CAPRI process must originate from reactions occurring in situ. Using in situ hydrogen generation reactions for the modeling of the THAI–CAPRI process within a simulation software, such as CMG STARS, has not yet been investigated, indicating that a novel approach to modeling THAI–CAPRI is necessary.

This study has highlighted the need for THAI–CAPRI to undergo numerical modeling using hydrogen generation reactions, such as water gas shift and coke gasification, and employs a novel method of modeling THAI–CAPRI within CMG STARS that includes hydrogen generation reactions such as those found within Kapadia, Kallos, and Gates^24^ rather than injected hydrogen. Using in situ-generated hydrogen for the catalytic upgrading of HO is to produce more accurate upgrading results and more representative of how the process would operate in situ. This will bridge the gap in knowledge of the practical process design between the laboratory testing of CAPRI and the field-scale simulation and possible pilot trial implementation of CAPRI within existing or new THAI projects worldwide.

## 2 Model Development

### 2.1 Initial THAI Model (Model T)

The original THAI model was built within the CMG Builder with data from the University of Nottingham and information from the model within.^5^ The model created and used within this paper is based on the aforementioned model, comprising 3990 evenly sized grid-blocks (30, 19, and 7 in the *X*, *Y*, and *Z*, respectively). The model contains eight components (Table 1) which participate in four reactions; one cracking reaction and three oxidation reactions (Table 2) under the operating parameters shown in Table 3.

**Table 1:** List of Components within the Lab-Scale THAI Model (Model T)^23^

component	critical pressure (kPa)	critical temperature (K)	molecular weight (kg mol^–1^)	density (×10^3^ kg cm^–3^)
water (H_2_O)	22,048	647.4	0.018	0.999
heavy oil (HO)	1031.29	1053.15	0.878	1.1075
light oil (LO)	2305.95	698.31	0.172	0.9038
CO_2_	7376	304.2	0.044	N/A
CO	3496	132.9	0.028	N/A
N_2_	3394	126.2	0.028	N/A
O_2_	5033.17	154.82	0.032	N/A
coke	N/A	N/A	0.013	N/A

**Table 2:** List of Reactions Found within the Lab-Scale THAI Model (Model T)^25^

	reaction	frequency factor (s^–1^)	activation energy (J mol^–1^)
1	HO → LO + coke	1.5 × 10^9^	0.99 × 10^5^
2	LO + O_2_ → H_2_O + CO_2_ + CO	1.812 × 10^12^	1.38 × 10^5^
3	HO + O_2_ → H_2_O + CO_2_ + CO	1.1812 × 10^11^	1.38 × 10^5^
4	coke + O_2_ → H_2_O + CO_2_ + CO	8.6 × 10^7^	1.23 × 10^4^

**Table 3:** Original Parameters Used within the Lab-Scale Model

parameter	value
air injection rate	8000 cm^3^ min^–1^
permeability *K*	3450 mD
permeability *I*and *J*	11,500 mD
porosity	0.34
initial pressure	200 kPa
initial temperature	27 °C

THAI involves an injection well, either vertical or horizontal, across the top of the producible fraction of the reservoir, and a horizontal producer. This model utilizes the horizontal injector due to gas override issues involving the vertical injector observed at this scale and mesh size. The injection well runs horizontally across the top layer (layer 1) on the left side (*i* = 1) of the model, while the production well runs vertically down the opposite side (*i* = 30) and horizontally across one of the bottom layers of the model until it terminates approximately 75% of the way across.

The injection well is heated for 30 min through an electrical heater within the wellbore before any injection occurs in a pre-injection heating cycle. At 30 min, air is injected into the model, at 21% oxygen, and initial combustion occurs. The reactions then proceed for another 290 min to generate comparable data with Greaves,^25^ at which point the composition of the products and plots of the ending parameters can be investigated. A diagrammatic representation has previously been presented.^5^

### 2.2 Model Augmentation for CAPRI (Model C)

The THAI model (model T) was supplemented with the addition of three new components as a novel aspect of this work; hydrogen, a hydro-treating catalyst, and upgraded HO components (Table 4). Furthermore, a catalyst pseudo-component was added to represent the catalyst in the THAI–CAPRI process. STARS does not have the capacity to directly model a catalyst, so a solid component (CAT) is used to represent the catalyst within the model. The CAT is included within the same horizontal blocks in which the producer is found at a concentration of 0.1 mol cm^–3^. Catalytic reactions are only able to take place where the CAT component is found representing the simplified behavior of a catalyst.

**Table 4:** List of Components within the Lab-Scale CAPRI Model (Model C)^29^

component	critical pressure (kPa)	critical temperature (K)	molecular weight (kg mol^–1^)	density (×10^3^ kg cm^–3^)
UHC	1523.46	775.34	0.253	0.85048
hydrogen (H_2_)	1315.50	33.44	0.002	N/A
catalyst (CAT)	N/A	N/A	0.013	1.0531

Details of the catalyst component details can be found in Hasan and Rigby,^23^ where a 44% packing porosity is employed. To represent the catalytic upgrading of the HO, a new hydrogenation reaction was introduced into the model (Reaction 8; Table 5). This reaction produced the new component upgraded heavy component (UHC) (Table 4), which represents an upgraded version of the HO component through a decrease in density and molecular weight.^26^ UHC is an adapted version of the upgraded component from Hasan and Rigby^23^ and is defined as a generalized HO component that has undergone hydrogen addition to decrease the density of the component. This reaction only took place where the catalyst pseudo-component occupied the cell, as the actual reaction would require the presence of a hydrotreating catalyst to occur. This was represented within STARS by having the CAT component on both sides of the reaction, as both a reactant and the product, with a ratio of 1:1 (Table 5).

**Table 5:** List of Reactions Found within the Lab-Scale CAPRI Model (Model C)^23,28^

	reaction	frequency factor (s^–1^)	activation energy (J mol^–1^)
5a	H_2_O + CO → H_2_ + CO_2_	5 × 10^9^	1.49 × 10^5^
5b	H_2_ + CO_2_ → H_2_O + CO	5 × 10^7^	1.9 × 10^5^
6	coke + H_2_O → CO + H_2_	2.12 × 10^12^	9.2 × 10^4^
7	coke + CO_2_ → CO	2.59 × 10^8^	5.4 × 10^4^
8	HO + H_2_ + CAT → UHC + CAT	8.5 × 10^18^	8.7 × 10^4^

#### 2.2.1 Grid Dependence

To investigate the grid convergence effects, less coarse meshes were trialed to aid in the resolution of the results, with models of up to 35,910 grid blocks (90 × 57 × 7) being used. However, due to the complexity of the proceeding CAPRI model (model C), less coarse meshes failed to converge before resulting in any meaningful data, often only reaching 1% progress after 24 h of run time, with large material balance errors. These issues have been attributed to the complexity and number of reactions within the CAPRI model, with large kinetic values (e.g., frequency factor) being used within the same equations as very small physical values (e.g., volume). 3990 is the number of grid blocks that allowed both models to have comparable results while minimizing the grid-block size as much as possible, also factoring in reasonable computing time.

### 2.3 Development of CAPRI Reactions

As previously stated, injecting hydrogen into an oil reservoir can lead to several risks, alongside economic disadvantages. Therefore, a paramount novel objective of this study is to model, using STARS, THAI–CAPRI without injecting hydrogen. Several mechanisms for hydrogen production are possible (e.g., methane reforming), but many encounter problems when under the conditions that are feasible within an oil reservoir, especially shallow HO/bitumen deposits due to low-pressure gradients and temperature limitations. In this study, water gas shift and coke gasification with water (coke steam reforming) have been selected (Reactions 5a and 6 from Table 5) as they are known to occur in the well.^18^ Oil upgrading is then achieved through the hydrodesulfurization, hydrodenitrogenation, and hydrocracking of the heavy end components within the in situ crude oil,^21,27^ represented in this study by the simplified hydroprocessing in Reaction 8 (Table 5).

#### 2.3.1 CAPRI Reaction Scheme

The reaction equations and kinetics for the CAPRI reactions are shown in Table 5. The kinetics for Reactions 5–7 were taken from Kapadia et al.^28^ and are all modeled as first-order reactions. Reaction 5 is split into two separate reactions (a and b), which both represent each direction of the reversible reaction. Reaction 8 is adapted from Hasan and Rigby^23^ through the removal of H_2_S and NH_2_ from the reaction for a simplified, all-encompassing version. Hydroprocessing of only the heavy end component occurs and not the lighter components, as reported by Greaves and Xia.^21^

### 2.4 Steam Variations for Model Investigations

Three different steaming variations are used for both THAI and THAI–CAPRI models to investigate the impacts of steam on the upgrading process (Table 6). All models have a 30 min pre-heating time before air injection occurs, with dry models (T1 and C1) utilizing electrical heaters around the injection wells to aid in oil ignition at a rate of 2115 J min^–1^^29^ Steamed models employ a combination of electrical heaters and steam for pre-heating.

**Table 6:** Steaming Variations for Models

model	steam protocol
T1	THAI + no steam
T2	THAI + 30 minpre-steam
T3	THAI + 780 min constant steam
C1	CAPRI + no steam
C2	CAPRI + 30 minpre-steam
C3	CAPRI + 780 min constant steam

### 2.5 CMOST Machine Learning Tool

CMOST is a machine learning tool within the CMG software suite. Selected model initialization input parameters are used as variables. CMOST then runs several models using these variables, varying the input values within a set range until a user-selected output result is reached. This study employs CMOST optimization as an automated validation tool. The selected parameters to carry out the optimization were the frequency factors within Tables 2 and 5. The reasons for this decision are two-fold: the exact kinetics experienced in situ are mostly unknown; and the reaction kinetics are varied by the user to overcome the issues associated with the grid volume(s) used within a simulation model. The range of values used for each variable was left as the suggested CMOST default range for consistency. Equations 1 and 2 were used to calculate the API at each time step for the produced oil
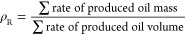
1
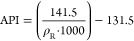
2

A constant of 1000 is used within eq 2 because oil density units were used in g cm^–3^. To find the average API, and therefore the average overall upgrading, the sum of the API for each time step was divided by the number of time steps.

#### 2.5.1 Model T

Initial CMOST inputs were obtained through published reaction kinetics within Greaves, Dong and Rigby^25^ (stoichiometry, frequency factor, and activation energy for Reactions 1–4). The optimization routine CMOST was set to run model C until a convergence criterion of an overall average API upgrading of ∼5° above the initial API was observed,^18^ which took 27 iterations.

#### 2.5.2 Model C

Initial CMOST inputs were obtained through published reaction kinetics within Greaves, Dong, and Rigby^25^ (Reactions 1–4), Kapadia et al.^28^ (Reactions 5a–7), and Hasan and Rigby^23^ (Reaction 8). The optimization routine CMOST was set to run model C until a convergence criterion of an overall average API upgrading of ∼12° above the initial API was observed,^18^ which took 32 iterations.

### 2.6 Model Validation

Both models have been successfully validated against published experimental data using CMOST. Model T was validated against two sets of published experimental data. Two variables are each compared against two separate published datasets, being API upgrading over time^8^ and peak temperature.^25^ Overall, the subsequent matches in the following sections are visually agreeable and show good similarity. Model C was validated against the data from Xia et al.,^18^ where an experiment was conducted using 10.5° API oil for THAI–CAPRI, and an upgrade of ∼11 to 22° API was noted. Addition of the catalyst in CAPRI causes an increase in the upgrading of about 5–7° API compared with only THAI, which is similar to the experimental observations of THAI–CAPRI versus THAI only as reported by Xia et al.^18^ and Greaves, Xia, and Nero.^15^ Since the model reported here contained new features, before investigating the effects of different variables, a base-case was validated by history matching to a set of experimental data gathered under similar conditions. Other studies of THAI–CAPRI oil upgrading in the lab produced an overall upgrading of API similar to that of Greaves, Xia, and Nero^15^ but varied in API versus time profile due to the variations in the experimental conditions. For this reason, the model could only be validated graphically against one study (Xia et al.^18^); however, the results are representative of experimental THAI–CAPRI results on the whole.

#### 2.6.1 Model T Peak Temperature

**Figure 1:**
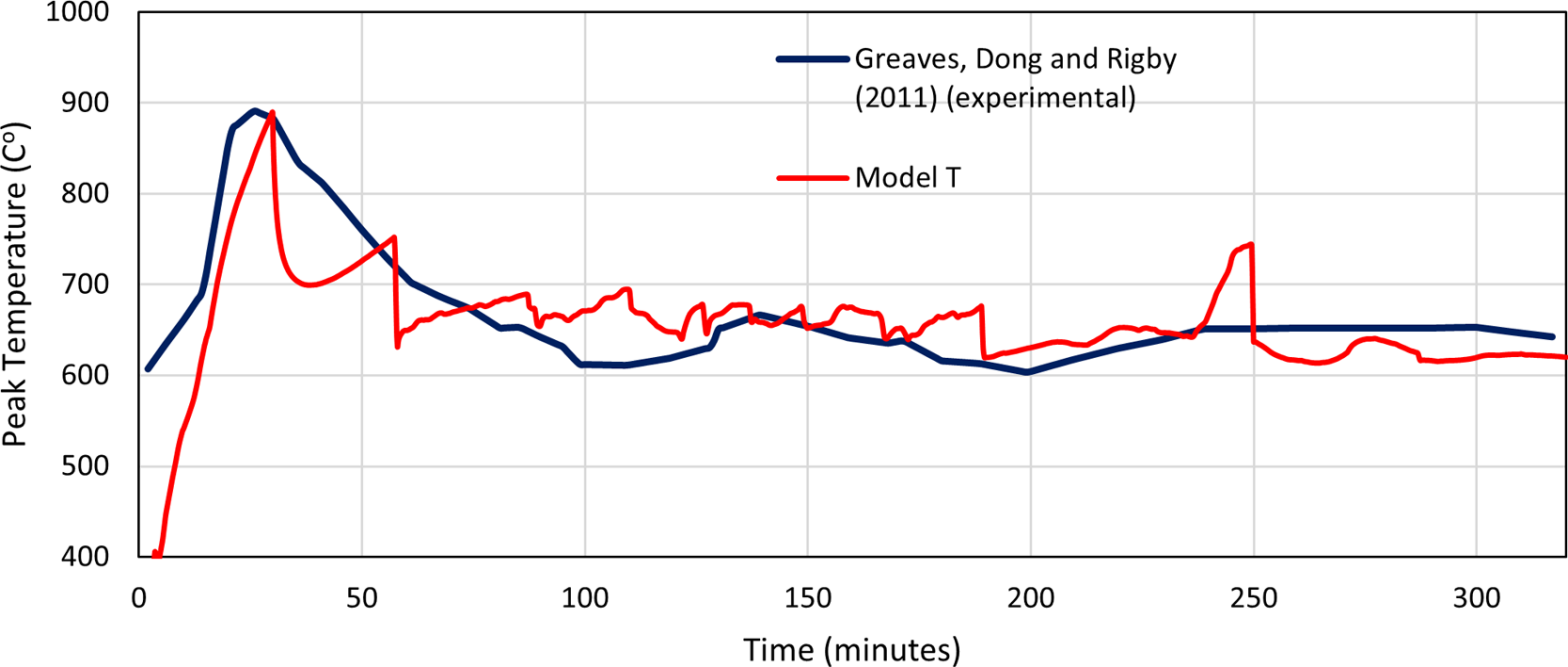
Peak temperature profile of model T against experimental data from Greaves, Dong, and Rigby.^25^

Both data sets display an initial rise to the peak temperature at 30 min, followed by a settling down to a stable, lower value of ∼200 to 250 °C less than the peak. The peak temperature is reached at the same time for both model T and the experimental work within Greaves, Dong, and Rigby,^25^ reaching the same temperature of just under 900 °C. However, there are some deviations within the profile throughout the 320 min, especially around 100 and 250 min. General deviations and undulations throughout could be explained by the low sampling rate and subsequent interpolation of the experimental temperature, in addition to the coarseness of the grid used in model T. However, the peak at 250 min is unexpected and as a result of the combustion of a large built-up area of coke within the coarse blocks of model T. Figure 1 shows the good agreement between the experimental results obtained from Greaves, Dong, and Rigby^25^ and compares them well to the simulated results from the same study.

#### 2.6.2 Model T API Matching

**Figure 2:**
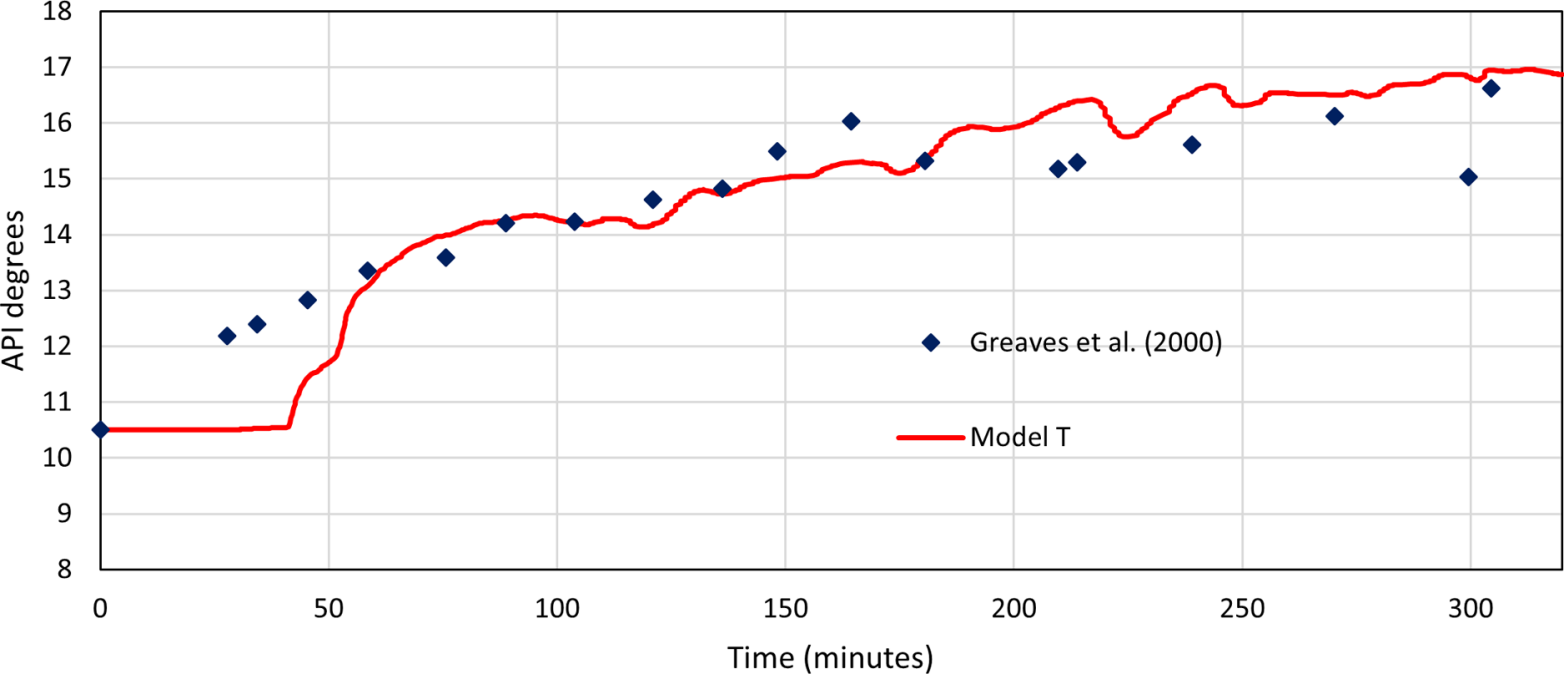
API degrees’ profile for model T against experimental data from Greaves and Xia.^16^

Model T was run with a starting API of 10.5° to represent the measure API of the Wolf Creek oil, which was used to validate against experimental work from Greaves et al.^8^Figure 2 shows the API of the produced oil against time for both data sets. The first 50 min display a large difference due to the low sampling rate of the experimental data combined with the start-up process of the simulation being impacted by more coarse grids. Visually, a good match is seen after 50 min for the API degrees of the produced oil over time, with the general trend and average values being extremely similar. Model T has a much higher variability, which again comes from a combination of the low sampling rates of the experimental data from Greaves et al.^8^ and the coarse grid used in model T.

#### 2.6.3 Statistical Modeling

API gravity similarities have been compared statistically for pseudo-*R*^2^ using python computer coding. *R*^2^ values fall between 0 and 1, with 1 being 100% matched and 0 being no match, respective to their linear trend. Moore^30^ states that an *R*^2^ value of 0.7 or higher indicates a strong correlation, and so 0.7 was used as the baseline for a good match within this study. However, fluctuations in the trends of the data are not represented, only the overall, overriding trend of the data. For this reason, data that visually match more closely could produce a lower score if the trend of the datasets fluctuates up and down over time. Different variables are compared to data from various sources due to a lack of full datasets within publications (e.g., no oil production rate data within Greaves, Dong, and Rigby).^25^

##### 2.6.3.1 Model T

Using the experimental data from Greaves et al.^8^ as the model data, model T is then compared against them for an *R*^2^ value to assess the similarity. Figure 2 has an *R*^2^ value of 0.89 and an API mean difference of 0.72. This value confirms that model T, when using consistent operational and model-development inputs as the experimental laboratory tests, is a very good match. Modeling laboratory-scale THAI processes in this approach produced accurate and valid results.

##### 2.6.3.2 Model C

**Figure 3:**
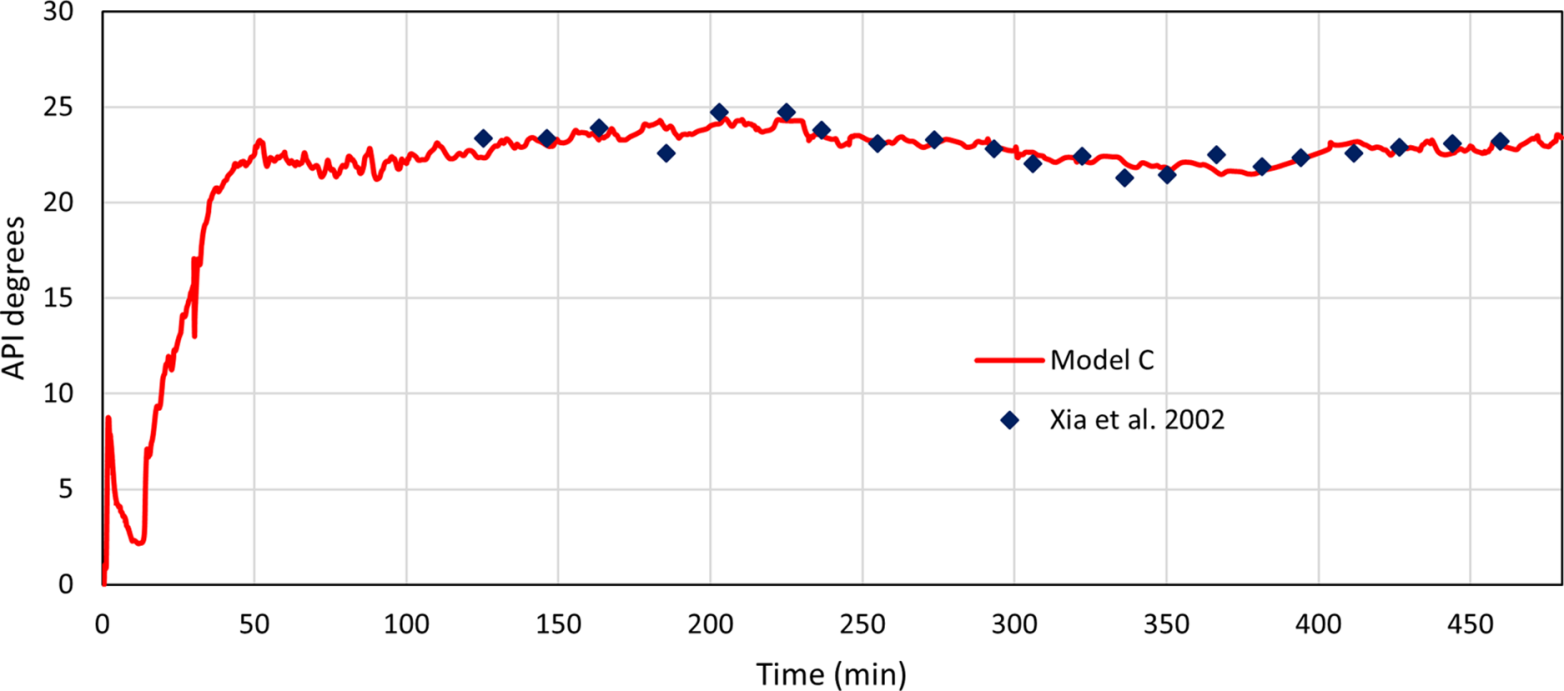
API of the produced oil vs time showing oil upgrading of model C against Xia et al.^18^

Using the experimental data from Xia et al.^18^ as the validation data, model C is then compared against them for an *R*^2^ value to assess the similarity. Figure 3 has an *R*^2^ value of 0.716 and an average API mean difference of 0.48. This value is lower than that seen for model T. However, due to the increased complexity of both the model as well as the data used for validation from Xia et al.,^18^ this decreased match is somewhat expected.

## 3 Results and Discussion

### 3.1 Temperature

#### 3.1.1 Peak Temperature

**Figure 4:**
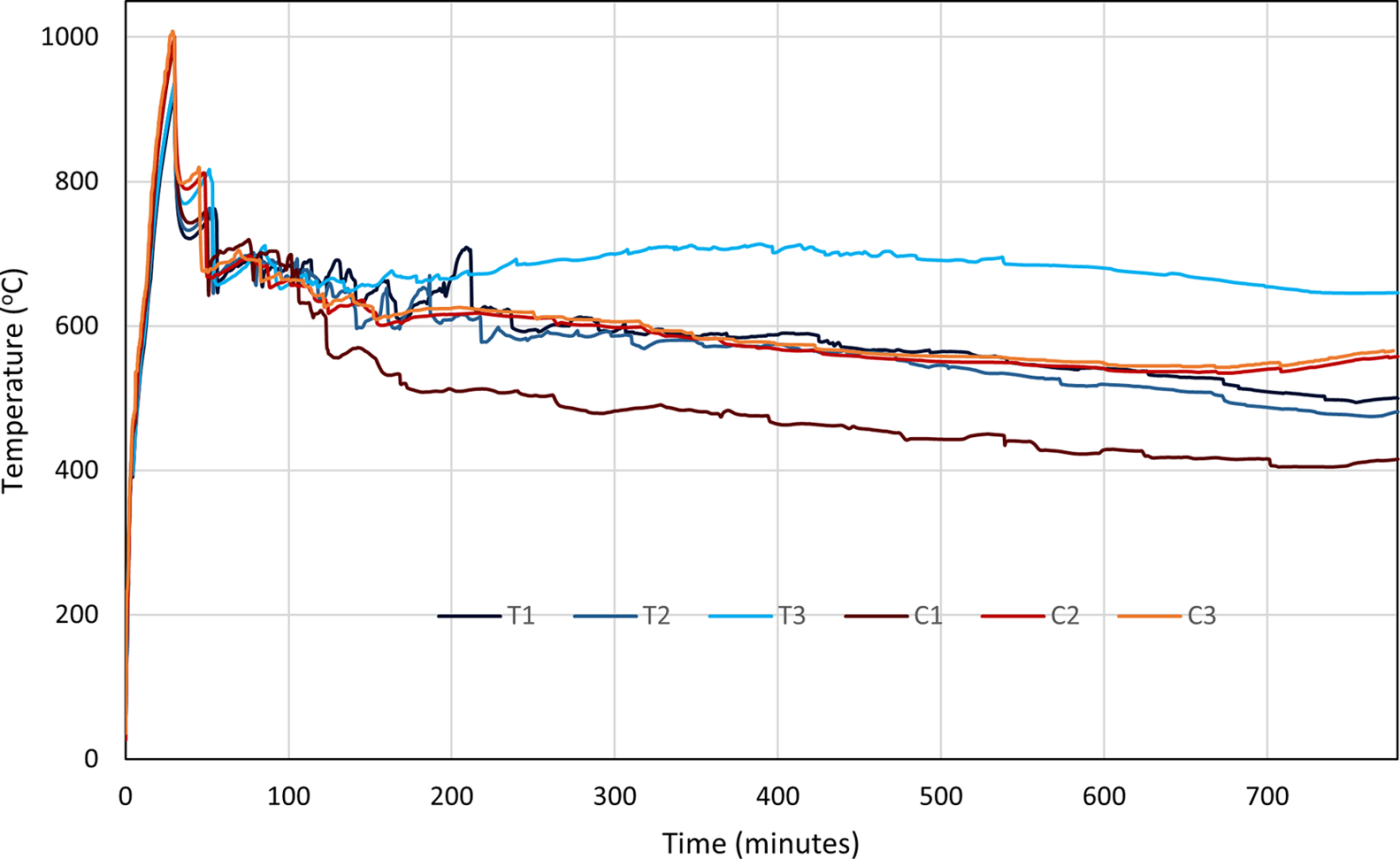
Temperature against time for THAI dry (T1), pre-steam (T2), and constant steam (T3) and THAI–CAPRI dry (C1) pre-steam (C2), and constant steam (C3).

The temperature calculated by model T can be observed to reach a peak of ∼900 °C after the injection of oxygen is initiated, with the initial temperatures being raised through the use of an electrical heater in the injector well, heating up the inlet area prior to oxygen injection. However, the temperature calculated by model C is observed to reach slightly higher, up to 1000 °C (Figure 4). The difference is explained through the additional electrical heating of model C to account for the endothermic coke gasification reactions. As the electrical heater raises the temperature of the inlet area, the thermal upgrade of the HO will begin, and it will crack into LO and coke. Due to the lack of oxygen injection at this time, the coke produced through this mechanism has no effect on the peak temperature of THAI, but in the CAPRI models, the coke is able to react with water (either native or injected) and bring the temperature down. As this occurs, additional electrical heating must be applied to the inlet area to maintain the peak temperature. The temperatures calculated by model T and model C both then show an undulating decrease in temperature down to ∼680 °C at 100 min. At 100 min, variations in the temperature start to become evident across the different models.

The temperatures of models T3 and C3 tend to level off or decrease gradually at ∼100 °C higher than those of models incorporating steaming between 100 min and 780 min. All model variations show a gradual decrease in the peak temperature from 150 min onward, except for model T3, which displays a slight increase until 400 min, at which point, the same decrease is then observed until 780 min.

#### 3.1.2 Temperature Distribution

**Figure 5:**
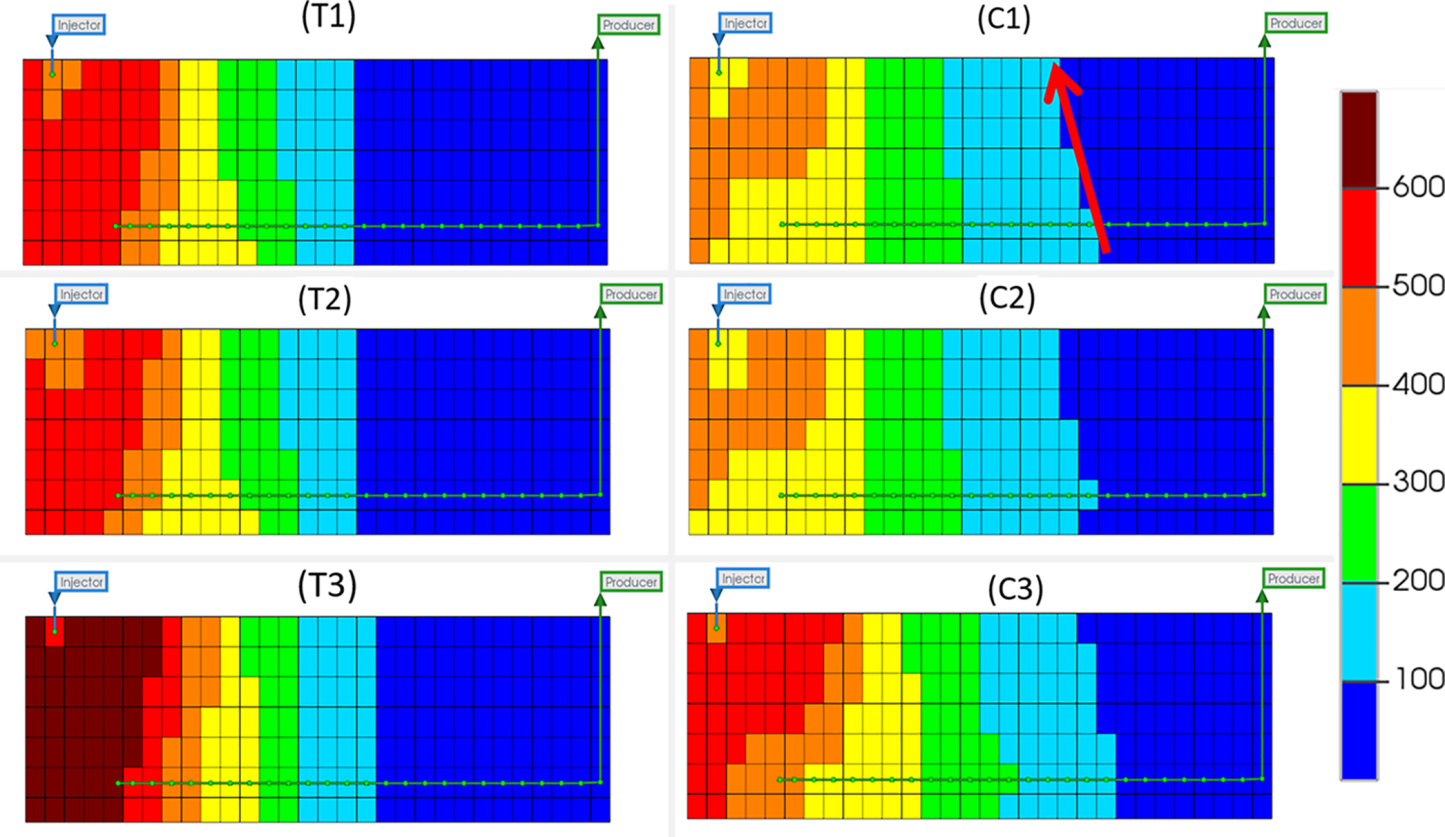
Temperature distribution (°C) for THAI dry (T1), pre-steam (T2), and constant steam (T3) and THAI–CAPRI dry (C1) pre-steam (C2), and constant steam (C3) at 320 min in the 10th *j* plane (scaled by a factor of 2 in the *z* direction).

**Figure 6:**
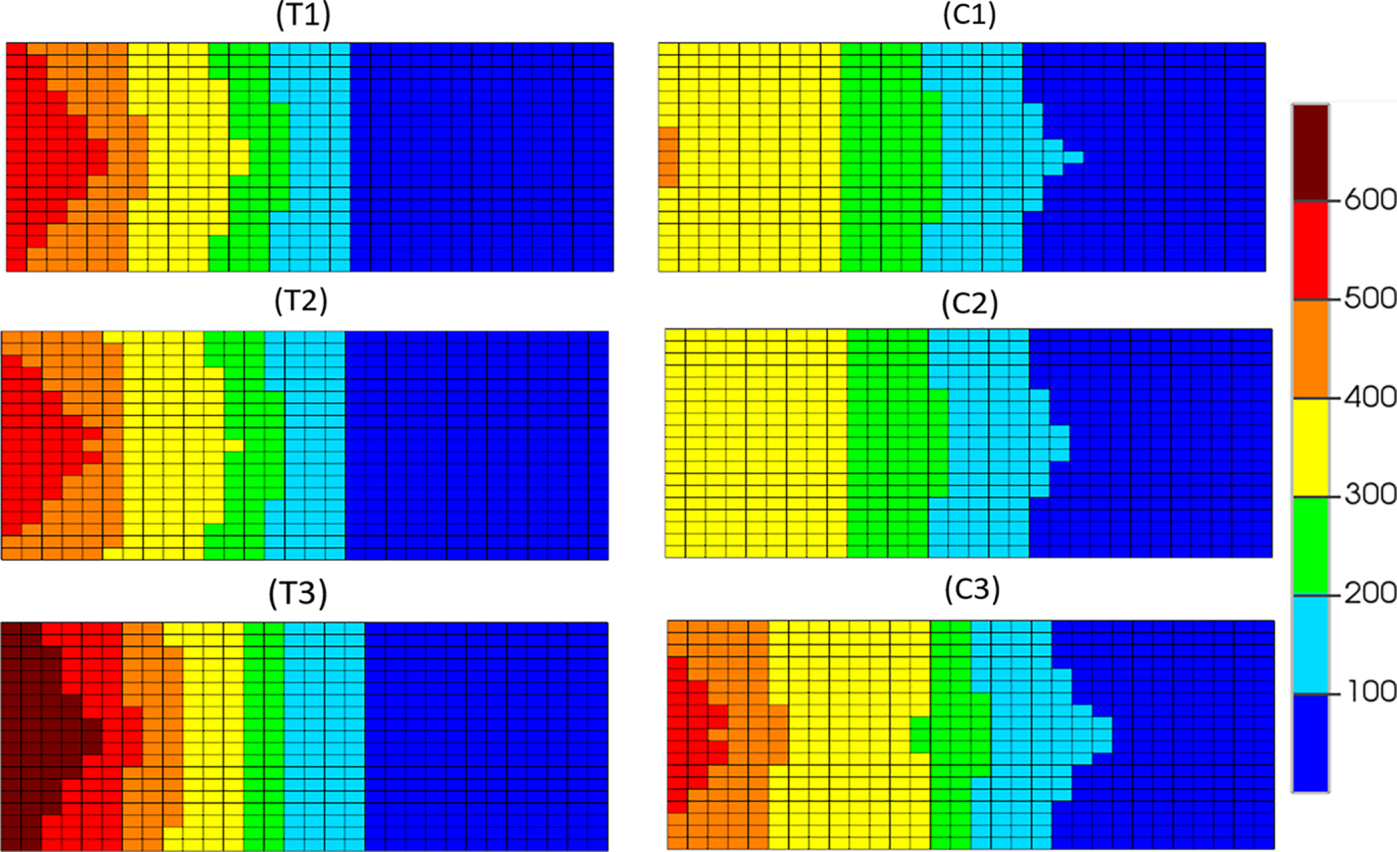
Temperature distribution (°C) for THAI dry (T1), pre-steam (T2), and constant steam (T3) and THAI–CAPRI dry (C1) pre-steam (C2), and constant steam (C3) at 320 min in the 6th *k* plane (scaled by a factor of 3 in the *i* direction).

Figure 5 shows the temperature distribution of all six models at 320 min, which is a point at which combustion has been initiated and the system should be in a stable process.^29^ Model T displays a forward leaning combustion front shown by the orange grids, with mostly vertical or forward-leaning contacts between all temperature regions, indicating that a stable combustion front has formed, and the sweep efficiency is high. A vertical or slightly forward-leaning combustion zone indicates a smooth moving area of combustion that consumes the oil from the sand-pack without causing effects such as channeling or gas overriding. Oil will also flow directly downward from the heated zone toward the producer well. Model C, however, displays a very prominently forward-leaning combustion front, with vertical or backward leaning contacts between all other temperature regions (as indicated by the red arrow). This indicates that the combustion front is somewhat unstable and may show poor performance in its ability to sweep the reservoir. These differences are caused by an increase in the temperature along the producer well of model C, shown by an elongation of the temperature regions toward the heel of the producer (also see Figure 6). This temperature increase will be as a direct result of exothermic catalysis in that area, with the higher temperatures caused by catalytic upgrading reactions of the HO causing a backward-leaning temperature gradient. This is most evident in the regions of lower temperature (200 °C+) and is concurrent with temperature profiles seen within experimental work from ref (18). The decrease in temperature calculated by model C when compared to model T is due to the lowered fuel availability.^19^ With the inclusion of coke gasification and HO hydrogenation within model C, there is competition of those components to be oxygenated, resulting in lower levels of combustion occurring and thus lower temperatures.

Figure 6 shows the temperature distribution of the models at 320 min for the plane in the downward (*k*) direction that contains the producer well at a cross section containing the injection and production wells. Little variation between the dry and pre-steamed variations of each model is observed, with differences between each dry and pre-steamed variation also being minimal when compared to the constant steam injection variations. Models T1 and T2 display more grid blocks at 400 °C or higher than model C1 and C2, which again is due to the lower fuel availability in that area as HO concentrations diminish more quickly in CAPRI as a result of additional reactions containing HO as a reactant. Pre-steaming the models appears to have a negligible impact on the temperature distribution seen throughout the models during stable combustion. This is expected as the additional steam from the start will have, by this point, joined the steam bank that has arisen ahead of the combustion front and will provide little to no additional help in heating the model. Constant steam injection, as it occurs in models T3 and C3, ensures that the producer well is maintained at a higher overall temperature for maximum thermal cracking and viscosity reduction of the HO in that area. These attributes are thought to increase the quality and quantity of the producer oil and demonstrated in the API upgrading observed in this study.

##### 3.1.2.1 Steam Impacts

The temperature of model C3 is seen to fall along the same time-series profile as those of models T1 and T2 (Figure 4) due to the increased heat provided by the constant steam injection. Model T3 reacts in the same way, producing a higher peak temperature and higher temperatures around the injector well than all other models due to the heat provided by the constant steam injection. It is believed that the temperature increase from the constant steam injection occurs dominantly as a result of physical heating of the process rather than being related to the exothermicity induced by the steam reacting. This is because none of the THAI models contain the reaction with steam/water as a reactant, yet still observes an increase in the peak temperature over those models with less or no steam.

##### 3.1.2.2 Impacts of the Mobile Oil Zone on Catalysis

Rabiu Ado^29^ states that simulations of the THAI process have shown that the mobile oil zone temperatures are not high enough for the catalysis of the HO hydrogenation to occur (300 °C); however, it can be seen that large areas of model T and model C are observed to be at least 300 °C, with most blocks surrounding the producer well in model C1, C2, and C3 being calculated to be 380 °C or higher, which is close to the optimum temperature of 425 °C observed in Hart and Wood.^14^ This suggests that catalysis is a feasible process that could occur within the THAI–CAPRI process within STARS through the addition of the catalyst component, though for optimum results in oil API upgrading, optimization of the operational processes that increase the temperature (e.g., increased air flux or oxygen enrichment) would be of benefit.

#### 3.1.3 Combustion Front

**Figure 7:**
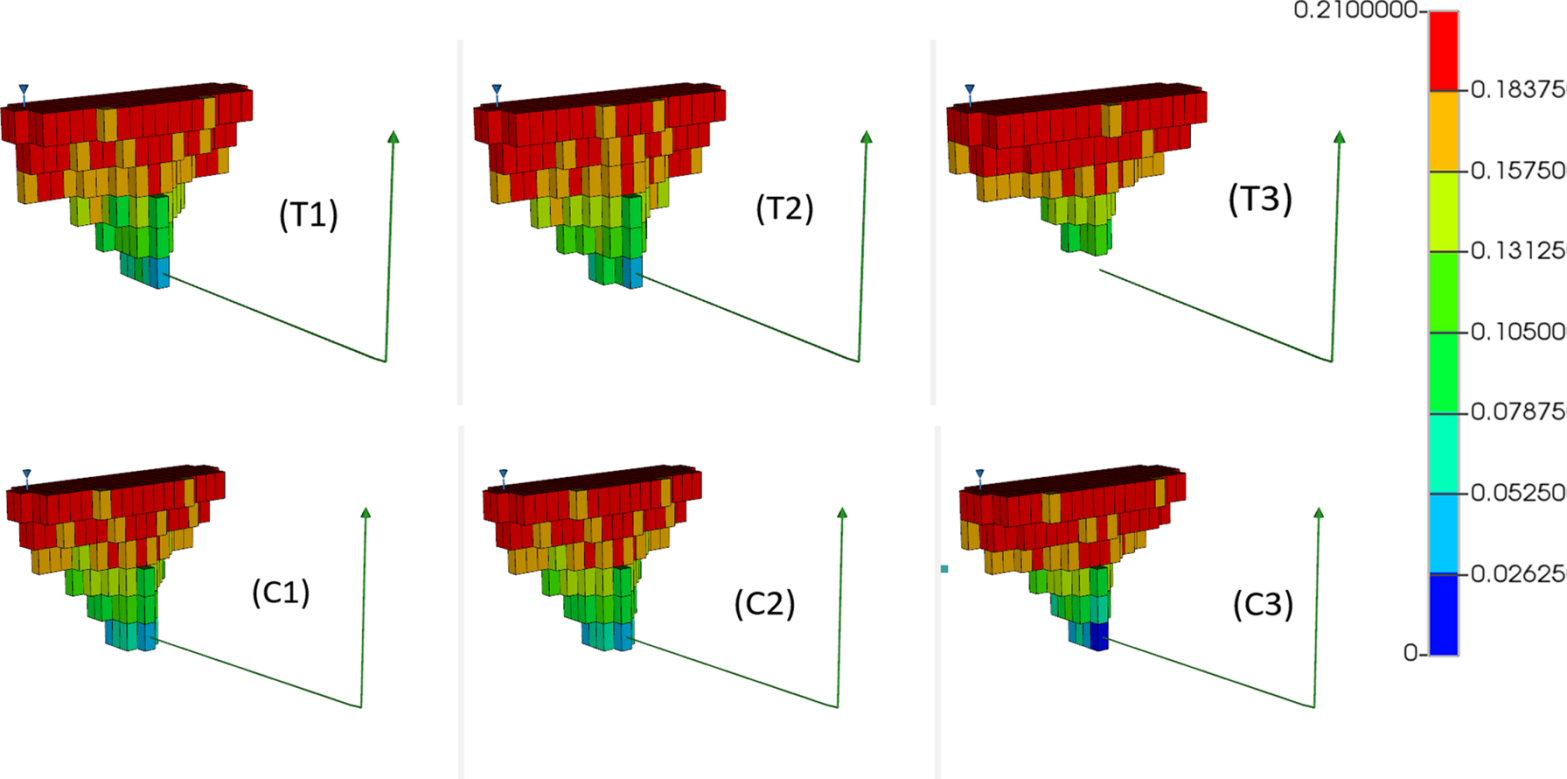
Oxygen gas mole fraction distribution for THAI dry (T1), pre-steam (T2), and constant steam (T3) and THAI–CAPRI dry (C1), pre-steam (C2), and constant steam (C3) at 320 min, showing only those grids that contain oxygen (scaled by a factor of 3 in the *i* direction).

During ISC, the region of the reservoir behind the combustion front is generally 100% saturated by gas, largely composed of unreacted oxygen.^29^ The shape of this oxygenated region should define the inner boundary of the combustion front as this is where oxygen begins to react with hydrocarbon fuels, bringing its saturation to zero; this region has been depicted in Figure 7 for the THAI and CAPRI models. Figure 7 shows the models in 3-D at 320 min, presenting only those grids that contain oxygen alongside the producer well. It is therefore an excellent indicator of the shape of the combustion front itself. By 320 min, the combustion front in all models has expanded to the full width of the model, gradually tapering down toward the toe of the producer (Figure 7). The distance between the toe of the producer and the bottom of the combustion front (i.e., the closest colored block to the producer well toe) varies from model to model, with model T3 displaying the largest value. All other models appear to display the combustion front contacting the producer well due the higher peak temperature of model T3 allowing for better oxygen consumption rates, allowing less oxygen to bypass the combustion zone unreacted. Unreacted oxygen reaching the production well is a key indication of early oxygen breakthrough;^19^ the lateral advancement of the combustion front is similar for all models, being 10 cm expansion at 320 min. This indicates an approximate combustion front velocity of 2 cm h^–1^, which is in line with the results observed in Xia et al.^31^ for experiments operated under a similar air injection flux.

### 3.2 Oil Upgrading

#### 3.2.1 American Petroleum Institute

**Figure 8:**
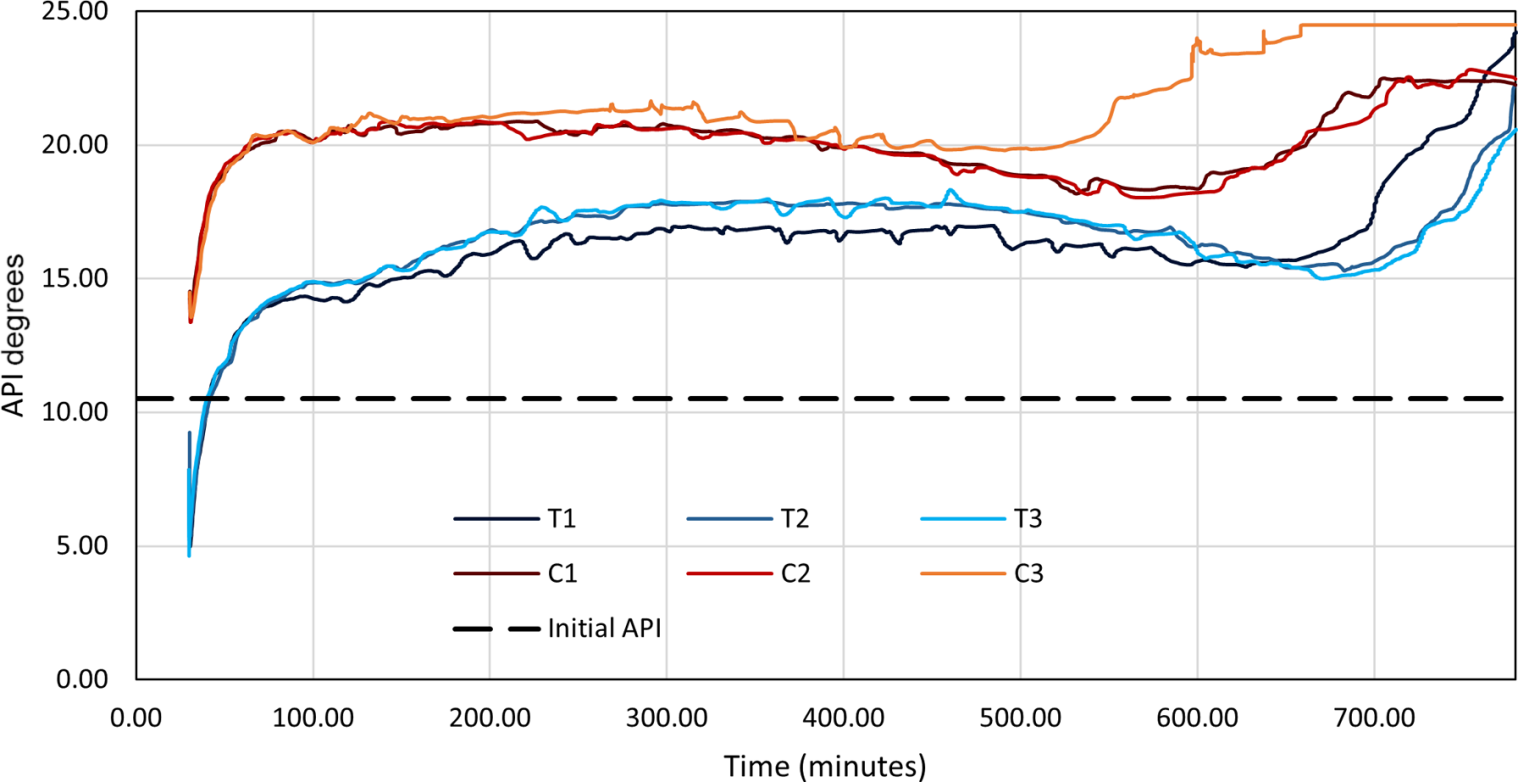
API against time for both THAI and THAI–CAPRI for the three investigated steam operating conditions. A black dashed line is used to represent the initial API of the oil before combustion.

**Figure 9:**
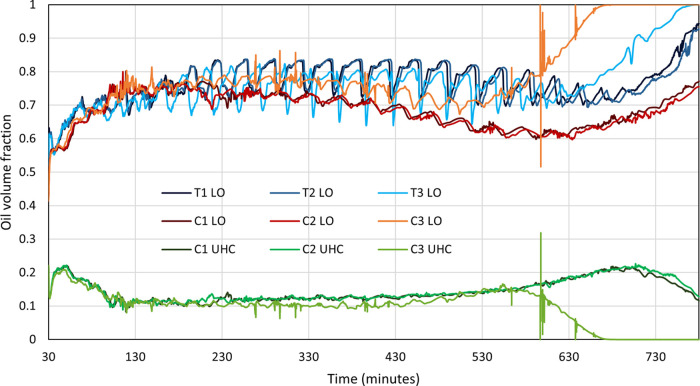
LO component oil volume fraction against time for both THAI and THAI–CAPRI for the three investigated steam operating conditions and the UHC component oil volume fraction for THAI–CAPRI for the three investigated steam operating conditions.

**Figure 10:**
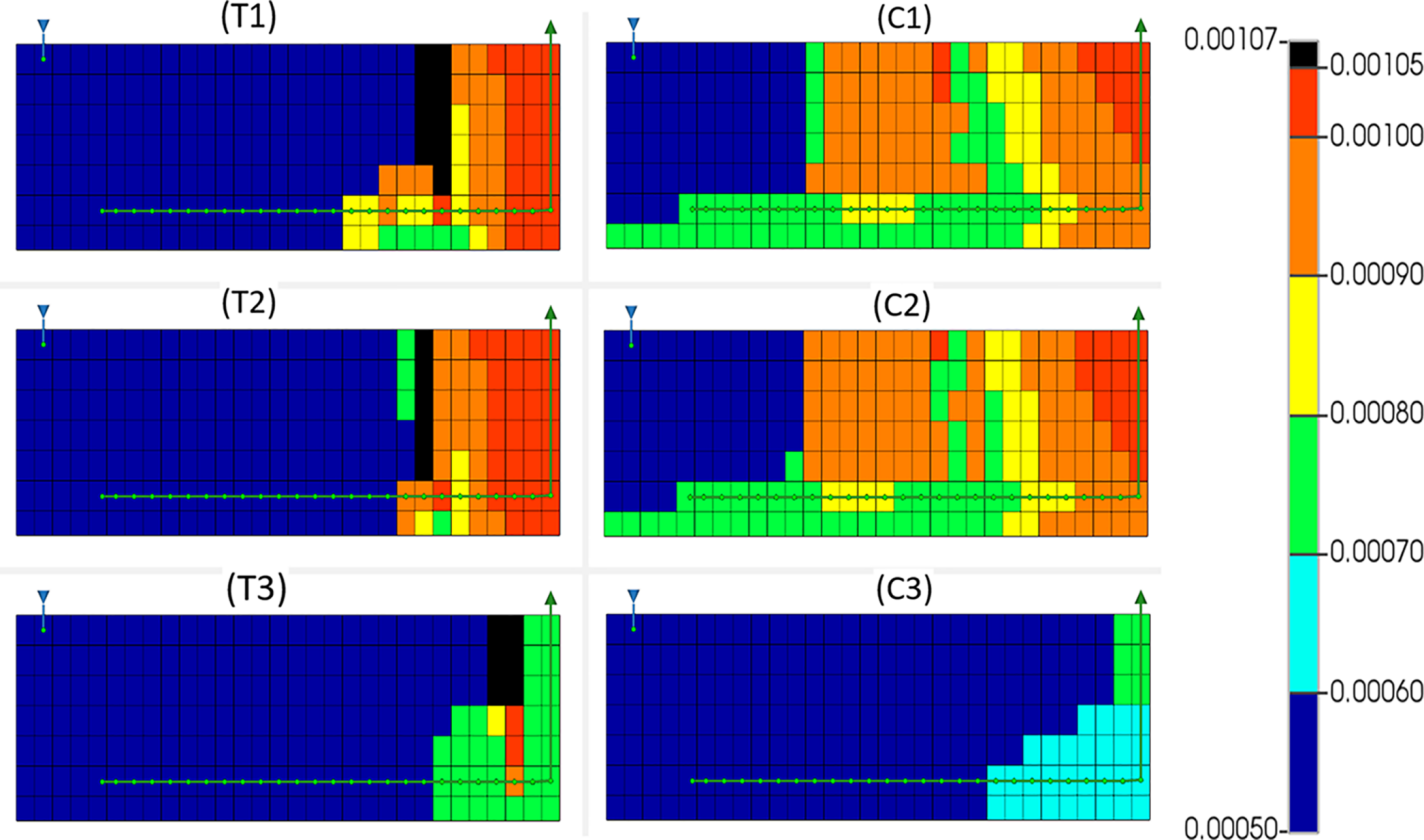
Oil density distribution (kg cm^–3^) for THAI dry (T1), pre-steam (T2), and constant steam (T3) and THAI–CAPRI dry (C1) pre-steam (C2), and constant steam (C3) at 780 min in the 10th *j* plane (scaled by a factor of 2 in the *z* direction).

The main use for both the THAI and THAI–CAPRI processes is the in situ upgrading of HOs and bitumen for the purpose of a lower-energy input route to fuel production. Figure 8 shows the API of the produced oil for dry, pre-steamed, and constant-steamed variations of both model T and model C as a function of the production time. All three THAI models display similar trends in API over time, showing a gradual increase and decrease over the first ∼600 min until sharp increases are observed in all three THAI models, to varying degrees. This is due to variations in the amount of lighter oil that builds up around the producer wellbore as a combined result of thermal cracking and gravity drainage due to a lowered density and viscosity. These lighter oils are therefore produced in these larger quantities at different times, with the higher temperatures of models C3 and T3 inducing higher rates of thermal cracking than those of models C1 and C2 and models T1 and T2, respectively, earlier within the 780 min run. Model T3 displays a lower API output than those of models T1 and T2 until 650 min when model T1 observes a sharp increase to ∼24.5° API, causing it to become higher than model T2 and T3, which also shows a lessened increase to ∼22 and ∼20° API, respectively. This eventual increase in API toward the end of the run is possibly explained through the comparable increase in LO production at that time (Figures 9 and 10). Model C3 displays a higher API output than those of models C1 and C2 at almost all times, indicating that increased steaming of model C causes higher amounts of HO to be upgraded. Model C is observed producing oil with an API of 2–5° higher than that of model T for most of the 780 min simulation run, implying that the presence of a catalyst does indeed aid in the in situ upgrading of HO through HO hydrogenation. These upgrading values for CAPRI over THAI are similar to the reported values under experimental conditions, averaging at 3.4, 5.8, and 6.3° API over THAI for no-steam, pre-steam, and constant-steam, respectively. However, this improvement varies over time, with API values for models T, C1, and C2 seemingly meeting by the end of the 780 min simulation run. This time-period is synchronous with a decrease in the temperature and could therefore indicate that higher degrees of API upgrading could occur through thermal cracking at lower temperatures due to the lower extent of high temperature oxidation (HTO) occurring. This is consistent with the reported results of Xia et al.,^18^ where it was found that API decreases during the time of temperature increase. The sharp decrease followed by the gradual increase in API at around 35 min in all six runs is attributed to the start-up effect. This consists of the LO present in the grids around the producer being produced before the oxidation process begins to occur during combustion.

#### 3.2.2 Produced Oil Composition

The ratio of oil components/fractions produced through both the THAI and THAI–CAPRI processes can be indicative of the extent and mechanism of upgrading (catalytic vs thermal cracking). To the authors’ best knowledge, the produced fractions of oil have not yet been investigated within STARS simulations of THAI and THAI–CAPRI. This novel analysis will shed light on the catalytic extent of CAPRI in lab-scale simulations. Figure 9 shows the volume fractions of the produced LO for all six models. The LO component production from models T1, T2, and T3 displays a significant undulation, which appears to be absent in the CAPRI models and API upgrading profiles for models T1, T2, and T3. This undulation could be indicative of unstable upgrading despite a stable combustion front being present, possibly caused by the use of a coarse gridding system within the model incrementally producing oil in larger quantities rather than consistently producing at a constant rate. This trend is absent in models C1, C2, and C3 due to the lower rate of thermal cracking occurring and lower temperatures leading to a lower decrease in viscosity of the LO fractions allowing for a slower, but more stable production. At ∼650 min, model C3 displays a plateau of LO production, reaching a 100% volume fraction, indicating that all HO, at least near the producer well, has been thermally cracked. This is concurrent with the volume fractions of the produced UHC shown in Figure 9, where at the same time, in model C3, UHC reaches zero due to no HO being available for catalytic upgrading as it has already all been thermally cracked. The same trends in increased LO and decreased UHC production in models C1 and C2 also occur concurrently for the same reasons that HO becomes less available for catalytic upgrading due to increased thermal cracking. Despite this, between ∼100 and ∼600 min, UHC production appears to be very stable in models C1, C2, and C3. These results are comparable to the interpretation of hydrogen to carbon (H/C) ratios and produced API within the literature of THAI and THAI–CAPRI laboratory experiments.^18^ However, laboratory experiments of THAI–CAPRI often show API upgrading and the H/C decline toward the end of the experiment after peaking.^14,18,21^ These declines are not observed in models C1, C2, and C3 in this investigation, being explained through the lack of catalyst deactivation within the STARS models.

#### 3.2.3 Oil Density Distribution

Figure 10 shows the oil density distribution, indicating that at 780 min, the average oil density for model C is lower than that of model T, with the black-colored grid squares representing oil that has experienced no change in density and colored squares representing oil of varying densities. It is also observed that although model T displays less grids containing oil of 0.0006 kg cm^–3^ or higher than model C, the average oil density visually remaining (determined by a higher proportion of grids containing oil of a lower density) within model T is higher than that of the oil remaining as calculated by model C at the same time. This is due to oil around the producer well in all model C variations displaying much lower average densities than the same blocks in all model T variations (represented by light-green and light-blue blocks), exhibiting the increased upgrading due to the catalyst. It should also be noted that the black grids displayed in models T1, T2, and T3 represent oil that has undergone no upgrading, indicating that certain regions of the model appear to be bypassed during the air injection process as a result of oxygen breakthrough creating override channels within the reservoir, suggesting that since the 320 min mark, stability has decreased within the process. Models T1 and T2 display oil density to generally decrease linearly from left to right, with the regions further away from the combustion front being higher in density. However, the same cannot be said for models C1 and C2; regions of the model undergo varying degrees of upgrading, consistent with decreasing temperature zones of the combustion front combined with disproportionate gravity segregation due to the augmented density decrease of oil around the production well, leading to heavier oils displacing less dense oil behind the combustion front, prompting an oil density distribution not observed in the catalyst-absent THAI models. Similar occurrences are observed within Ado, Greaves, and Rigby.^19^ Constantly steaming the process appears to have the biggest impact on decreasing the density of the in situ oil, with model C3 displaying all residual oil to be less than 0.0008 kg cm^–3^, or 45° API. The results of model T3 show some similarity to those of model C3 in increased density decrease through constant steam injection. However, it appears to still have regions of unreacted or less significant decreases in density due to the absence of catalytic upgrading within the model.

### 3.3 Coke Production

**Figure 11:**
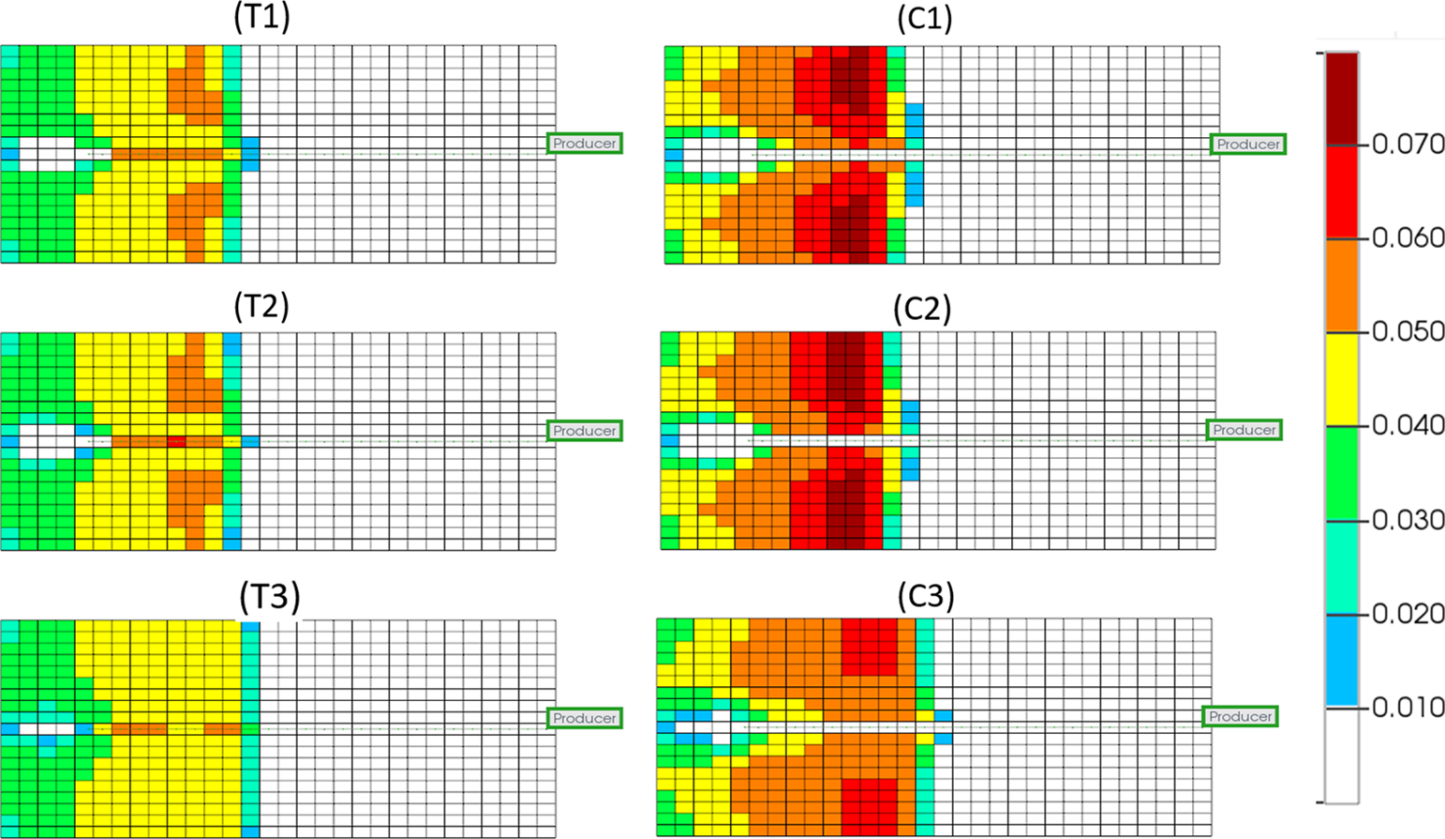
Coke concentration (mol cm^–3^) for THAI dry (T1), pre-steam (T2), and constant steam (T3) and THAI–CAPRI dry (C1), pre-steam (C2), and constant steam (C3) at 320 min in the 6th *k* plane (scaled by a factor of 3 in the *i* direction).

Coke is deposited during thermal cracking of HO and occurs in the region just ahead of the combustion front and is primarily used as fuel for the advancing combustion front.^10,29^ Coke that is observed to be behind the combustion front is HTO fuel that has been bypassed by the advancing combustion front, indicating that perhaps higher air injection rates are required for complete combustion of coke fuel. This bypassing is observed in all six models (Figure 11). Also observed is the regions of higher coke concentration as indicated by regions of darker orange/red shaded grid squares. These areas are the symptomatic of the coking front found just ahead of the combustion front where thermal cracking occurs, displaying a relationship between coke concentration and combustion front shape. In models T1, T2, and T3 more coke is observed in the grids where the horizontal producer well is located, suggesting that coke that is laid down on the well is more likely to be bypassed by the advancing combustion front. Conversely, models C1, C2, and C3 display the same grids containing negligible coke within them. However, this is thought to be an artifact of the simulator not depositing solid coke component in the grids where the solid catalyst component is located. A relationship between coke concentration and peak temperature can also be seen when Figures 11 and 6 are compared. This is particularly evident through the decrease in coke concentration in models T3 and C3 when compared to their lesser steamed counterparts due to increased coke consumption leading to higher temperatures, which in turn also accelerates the coke consumption. Interestingly, models C1, C2, and C3 display significantly higher concentrations of coke than their non-catalyzed counterparts in THAI despite having additional coke consumption reactions via coke gasification. This is possibly explained through lower temperatures leading to much lower HTO rates of coke,^18,32^ allowing more coke to be bypassed by the combustion front than in models T1, T2, and T3 while already having been bypassed by the steam bank reducing the coke gasification of the combustion bypassed coke.

### 3.4 Oil Production

**Figure 12:**
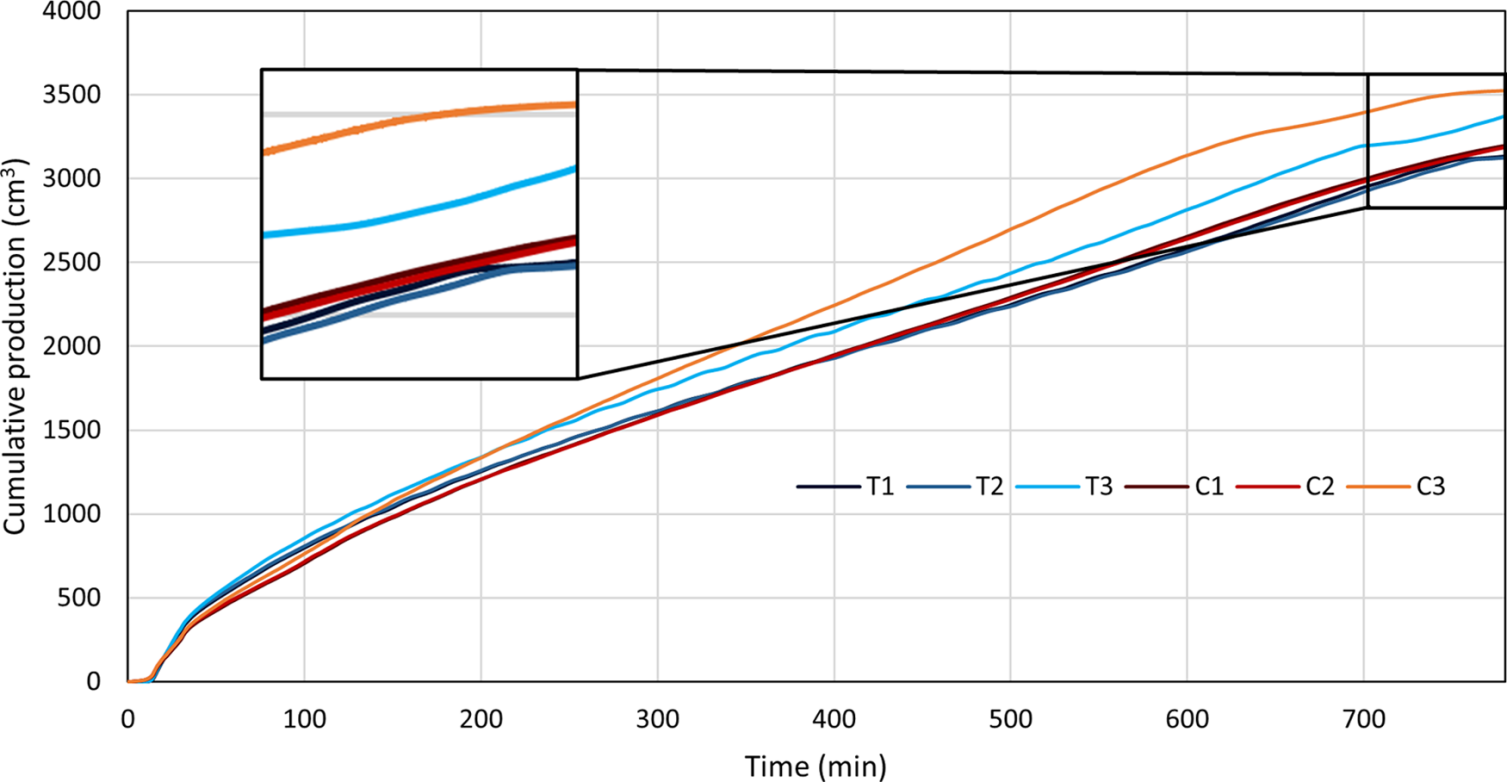
Cumulative oil production against time for both THAI and THAI–CAPRI for the three investigated steam operating conditions.

Figure 12 shows cumulative oil production from all THAI and CAPRI models. A negligible difference is observed between any of the no-steam and pre-steamed models, with model C1 displaying the highest cumulative production of the four (Figure 12). However, constant steaming of the models, as incorporated into models T3 and C3, results in significantly higher cumulative production, with model T3 and model C3 experiencing an increase of ∼200 and 400 cm^3^, respectively, above ∼3150 cm^3^ of the other four models. This is concurrent with thermal cracking, which occurs in models T3 and C3, converting most, if not all, HO into LO, which has a much lower viscosity, allowing for easier flow and production. A larger increase in cumulative production is displayed in model C3 due to the increased proportion of HO that is converted into UHC, which again has a much lower viscosity than HO, allowing for more efficient production of the oil. The above factors and higher temperature associated with constant steaming lead to much lower viscosities of all oil components and increased cumulative production. Very little variation between models T1 and T2 is seen; similarly, negligible variation occurs between models C1 and C2, suggesting that pre-steaming of these processes does little to increase the production potential. It should also be noted that models C1 and C2 display very little increase in production over models T1 and T2, implying that the addition of a catalyst to the THAI process does little to increase the cumulative production unless constant steaming of the process is applied, consistent with Xia et al.^18^ This does not, however, account for the quality of the produced oil, which will still be higher and more favorable in the THAI–CAPRI process.

### 3.5 Comparison to Hydrogen Injection

Modeling THAI–CAPRI using the co-injection of oxygen and hydrogen can provide a good idea of the potential upgrading of the process. It can suggest an optimal oxygen to hydrogen ratio for the operation of THAI–CAPRI, offering optimum hydrogen concentrations for oil upgrading under THAI conditions. Several papers have published results of such investigations^32^ citing similar upgrading potential to the models within this study. However, the inclusion of hydrogen generation reactions leads to differing results for fuel availability, temperature, and oil production. The inclusion of coke gasification in model C leads to less fuel availability and therefore less HTO of coke. This results in a lower calculated temperature, which is exacerbated by the endothermicity of the coke gasification reaction. These lower temperatures are realized in the oil production where the cumulative oil production for model C1 in Figure 12 at 320 min sits approximately 450 cm^3^ less than the cumulative oil production from Ado, Greaves, and Rigby^32^ at the same time. The lower temperatures lead to decreased viscosity reduction and thermal cracking, causing a reduced flow potential of the oil. Hydrogen injection within THAI–CAPRI results in an overestimation of the absolute cumulative oil production.

Ado, Greaves, and Rigby^32^ also used a minimum activation temperature (*T*_a_) of 400 °C for the catalytic hydrogen addition reaction, meaning that if a grid block containing all the necessary reactants was below *T*_a_ then 400 °C would be used within the Arrhenius equation to calculate the reaction rate. However, Ado, Greaves, and Rigby^32^ used the same activation energy as this study, with both investigations reporting similar oil upgrading when compared to THAI, yet this study did not use a *T*_a_. Not using a *T*_a_ would lead to more accurate density distributions as only those areas that are at the correct conditions will demonstrate catalytic oil upgrading. This study also demonstrates that if a CAPRI model uses the activation energy from Ado, Greaves, and Rigby^32^ then catalytic upgrading is possible at the temperatures within the THAI process without the need of using a *T*_a_.

### 3.6 Comparison of Models

Overall, all six models experienced an increase in API over the original oil of at least 58.76%. Constant steaming of the THAI and THAI–CAPRI process increases oil production by at least 7.7 and 10.6% over the dry variations, respectively (Table 7). Model C3 showed the greatest API upgrading and cumulative oil production when compared to all other models, with model T3 showing the second largest increase in cumulative oil and model C2 the second largest increase in API upgrading. The utilization of either or both constant steaming and a producer well loaded with a catalyst would be dependent on the needs of the process. It is evident that even without the use of any steaming, CAPRI can significantly upgrade in situ HO. However, CAPRI has been shown not to increase oil production over THAI until constant steaming is exploited, with constant steaming of THAI performing well in increasing oil production also, at the cost of no increased API upgrading over dry THAI. Overall, the results have shown that for both THAI and THAI–CAPRI, pre-steaming has negligible impacts due to the timing of the steam bank formation.

**Table 7:** Comparison of End-of-Run Results^a^

model number	average produced API (deg)	% increase over initial API	cumulative produced oil (cm^3^)	% increase in cumulative oil over T1^+^/C1*
T1	16.74	59.43	3124.38	N/A
T2	16.67	58.76	3117.92	–0.21^+^
T3	16.77	59.71	3365.89	7.73^+^
C1	20.14	91.81	3184.71	N/A
C2	22.49	114.19	3173.35	–0.36*
C3	23.08	119.81	3524.37	10.67*

a + indicates % increase in cumulative oil over model T1. * indicates % increase in cumulative oil over model C1.

## 4 Conclusions

Simulation of experimental THAI and THAI–CAPRI processes is possible within CMG STARS and can readily lead to the ability to investigate phenomena related to reaction kinetics and operation conditions. The THAI–CAPRI process has also been modeled using in situ-produced hydrogen and has been shown to offer potentially a more accurate oil upgrading behavior of in situ HO. Validated models of both processes display both good matches through visual comparison and statistical *R*^2^ analysis (0.89 and 0.72 for THAI and CAPRI for API upgrading, respectively). Both models in this study were validated and matched against experimental data taken from the extant literature, with CMG CMOST machine learning being a valuable tool in the validation through comparing and matching of simulated and experimental data, respectively. This study investigated the effects of various steaming protocols on the THAI and THAI–CAPRI processes through numerical modeling and simulation. Three protocols were used: no steam injection, 30 min pre-steam, and 780 min constant steam. Overall, constant steaming of the processes displayed the highest quality of oil produced alongside the higher cumulative production of oil. THAI–CAPRI was found to be favorable for API upgrading irrelevant of the steaming protocol used. However, constantly steaming the THAI–CAPRI process resulted in the highest amount of API upgrading of both the in situ and produced oil. Overall, THAI–CAPRI experienced an increase in average API upgrading of 3.4, 5.8, and 6.3 over that of THAI for no-steam, pre-steam, and constant-steam, respectively. Cumulative production rates varied from ∼3150 to ∼3500 cm^3^, with constant steaming of the process increasing production the most through improved oil viscosity reduction.

## References

[ref1] Hein F. J. Geology of bitumen and heavy oil: An overview. J. Pet. Sci. Eng. 2017, 154, 551–563. 10.1016/j.petrol.2016.11.025.

[ref2] Kothari R.; Buddhi D.; Sawhney R. L. Comparison of environmental and economic aspects of various hydrogen production methods. Renewable Sustainable Energy Rev. 2008, 12, 553–563. 10.1016/j.rser.2006.07.012.

[ref3] Fujimori S.; Su X.; Liu J.-Y.; Hasegawa T.; Takahashi K.; Masui T.; Takimi M. Implication of Paris Agreement in the context of long-term climate mitigation goals. SpringerPlus 2016, 5, 2118 10.1186/s40064-017-3787-3. 28820915 PMC5451371

[ref4] Singh A.; Mathewes T.; Dalawat K.; Agarwal J.; Shah M. THAI-CAPRI Technology for Heavy Crude Reserves. Twelve International Conference on Thermal Engineering: Theory and Applications, 2019.

[ref5] Ado M. R.; Greaves M.; Rigby S. P. Numerical simulation of the impact of geological heterogeneity on performance and safety of THAI heavy oil production process. J. Pet. Sci. Eng. 2019, 173, 1130–1148. 10.1016/j.petrol.2018.10.087.

[ref6] Jimenez J. The Field Performance of SAGD Projects in Canada. In International Petroleum Technology Conference; IPTC-12860-MS, 2008.

[ref7] Moore R. G.; Laureshen C. J.; Belgrave J. D. M.; Ursenbach M. G.; Mehta S. A. R. In situ combustion in Canadian heavy oil reservoirs. Fuel 1995, 74, 1169–1175. 10.1016/0016-2361(95)00063-b.

[ref8] Greaves M.; Xia T. X.; Turta A. T.; Ayasse C. Recent Laboratory Results of THAI and its Comparison with Other IOR Processes. SPE/DOE Improved Oil Recovery Symposium, 2000.

[ref9] Jinzhong L.; Wenlong G.; Yongbin W.; Bojun W.; Jihong H. Combustion Front Expanding Characteristic and Risk Analysis of THAI Process. IPTC 2013: International Petroleum Technology Conference, 2013; Vol. 1.

[ref10] Xia T. X.; Greaves M.; Turta A. Main Mechanism for Stability of THAI- “Toe-to-Heel Air Injection”. Canadian International Petroleum Conference, 2003.

[ref11] Shah A.; Fishwick R.; Wood J.; Leeke G.; Rigby S.; Greaves M. A review of novel techniques for heavy oil and bitumen extraction and upgrading. Energy Environ. Sci. 2010, 3, 700–714. 10.1039/b918960b.

[ref12] Turta A.; Greaves M.; Grabovski J. Comprehensive Assessment of Toe-To-Heel Air Injection (THAI) Process. Guidelines for Development of Future Generations of In-Situ Combustion Processes. In Report Issued by AT EOR Consultancy, Calgary, January, 2018.

[ref13] Ayasse C.; Bloomer C.; Lyngberg E.; Boddy W.; Donnelly J.; Greaves M. First Field Pilot of the THAI Process. Canadian International Petroleum Conference, 2005.

[ref14] Hart A.; Wood J. In situ catalytic upgrading of heavy crude with CAPRI: influence of hydrogen on catalyst pore plugging and deactivation due to coke. Energies 2018, 11, 636 10.3390/en11030636.

[ref15] Greaves M.; Xia T. X.; Imbus S.; Nero V. THAI-CAPRI Process: Tracing Downhole Upgrading of Heavy Oil. Canadian International Petroleum Conference, 2004.

[ref16] Greaves M.; Xia T. X. Simulation Studies of THAI Process. Canadian International Petroleum Conference, 2000.

[ref17] Greaves M.; Xia T. X.; Turta A. T. Stability of THAI process-Theoretical and experimental observations. J. Can. Pet. Technol. 2008, 47 (). 10.2118/08-09-65

[ref18] Xia T.; Greaves M.; Werfilli W.; Rathbone R. Downhole Conversion of Lloydminster Heavy Oil Using THAI-CAPRI Process. SPE International Thermal Operations and Heavy Oil Symposium and International Horizontal Well Technology Conference, 2002.

[ref19] Ado M. R.; Greaves M.; Rigby S. P. Effect of operating pressure on the performance of THAI-CAPRI in situ combustion and in situ catalytic process for simultaneous thermal and catalytic upgrading of heavy oils and bitumen. Pet. Res. 2022, 7, 155–164. 10.1016/j.ptlrs.2021.09.010.

[ref20] Greaves M.; Xia T. CAPRI-Downhole Catalytic Process for Upgrading Heavy Oil: Produced Oil Properties and Composition. Canadian International Petroleum Conference, 2001.

[ref21] Greaves M.; Xia T. Downhole upgrading of Wolf Lake oil using THAI–CAPRI processes-tracer tests. Prepr. Pap.-Am. Chem. Soc., Div. Fuel Chem. 2004, 49, 69–72.

[ref22] Hart A. The novel THAI–CAPRI technology and its comparison to other thermal methods for heavy oil recovery and upgrading. J. Pet. Explor. Prod. Technol. 2014, 4, 427–437. 10.1007/s13202-013-0096-4.

[ref23] Hasan M.; Rigby S. Enhanced Recovery of Heavy Oil Using A Catalytic Process. IOP Conf. Ser.: Mater. Sci. Eng. 2019, 579, 012030 10.1088/1757-899X/579/1/012030.

[ref24] Kapadia P. R.; Kallos M. S.; Gates I. D. Potential for hydrogen generation from in situ combustion of Athabasca bitumen. Fuel 2011, 90, 2254–2265. 10.1016/j.fuel.2011.02.038.

[ref25] Greaves M.; Dong L.; Rigby S. Upscaling THAI: Experiment to pilot. Canadian Unconventional Resources Conference; Society of Petroleum Engineers, 2011; Vol. 2.

[ref26] Escobar J.; Barrera M. C.; Santes V.; Terrazas J. E. Naphthalene hydrogenation over Mg-doped Pt/Al2O3. Catal. Today 2017, 296, 197–204. 10.1016/j.cattod.2017.04.064.

[ref27] Ferdous D.; Dalai A. K.; Adjaye J. Hydrodenitrogenation and hydrodesulfurization of heavy gas oil using NiMo/Al2O3 catalyst containing boron: Experimental and kinetic studies. Ind. Eng. Chem. Res. 2006, 45, 544–552. 10.1021/ie050094r.

[ref28] Kapadia P. R.; Wang J. J.; Kallos M. S.; Gates I. D. Practical process design for in situ gasification of bitumen. Appl. Energy 2013, 107, 281–296. 10.1016/j.apenergy.2013.02.035.

[ref29] Rabiu Ado M. Numerical simulation of heavy oil and bitumen recovery and upgrading techniques. Doctoral Dissertation, University of Nottingham, 2017.

[ref30] Moore L. M. The Basic Practice of Statistics; Taylor & Francis, 1996.

[ref31] Xia T.; Greaves B.; Werfilli M. S.; Rathbone R. R. THAI Process-Effect of Oil Layer Thickness on Heavy Oil Recovery. Canadian International Petroleum Conference, 2002.

[ref32] Ado M. R.; Greaves M.; Rigby S. P. Simulation of catalytic upgrading in CAPRI, an add-on process to novel in-situ combustion, THAI. Pet. Res. 2022, 7, 297–307. 10.1016/j.ptlrs.2021.10.002.

